# Methane formation driven by light and heat prior to the origin of life and beyond

**DOI:** 10.1038/s41467-023-39917-0

**Published:** 2023-08-01

**Authors:** Leonard Ernst, Uladzimir Barayeu, Jonas Hädeler, Tobias P. Dick, Judith M. Klatt, Frank Keppler, Johannes G. Rebelein

**Affiliations:** 1 Max Planck Institute for Terrestrial Microbiology, 35043 Marburg, Germany; 2 Center for Synthetic Microbiology (SYNMIKRO), 35032 Marburg, Germany; 3 Division of Redox Regulation, German Cancer Research Center (DKFZ), DKFZ-ZMBH Alliance, 69120 Heidelberg, Germany; 4 Faculty of Biosciences, Heidelberg University, 69120 Heidelberg, Germany; 5 Institute of Earth Sciences, Heidelberg University, 69120 Heidelberg, Germany; 6 Microcosm Earth Center, Max Planck Institute for Terrestrial Microbiology & Philipps University Marburg, 35032 Marburg, Germany; 7 Biogeochemistry Group, Department for Chemistry, Philipps University Marburg, 35032 Marburg, Germany; 8 Heidelberg Center for the Environment HCE, Heidelberg University, 69120 Heidelberg, Germany

**Keywords:** Carbon cycle, Carbon cycle

## Abstract

Methane is a potent greenhouse gas, which likely enabled the evolution of life by keeping the early Earth warm. Here, we demonstrate routes towards abiotic methane and ethane formation under early-earth conditions from methylated sulfur and nitrogen compounds with prebiotic origin. These compounds are demethylated in Fenton reactions governed by ferrous iron and reactive oxygen species (ROS) produced by light and heat in aqueous environments. After the emergence of life, this phenomenon would have greatly intensified in the anoxic Archean by providing methylated sulfur and nitrogen substrates. This ROS-driven Fenton chemistry can occur delocalized from serpentinization across Earth’s humid realm and thereby substantially differs from previously suggested methane formation routes that are spatially restricted. Here, we report that Fenton reactions driven by light and heat release methane and ethane and might have shaped the chemical evolution of the atmosphere prior to the origin of life and beyond.

## Abstract (web summary)

Abiotic methane and ethane formation routes in aqueous environments driven by light and heat are identified. The released hydrocarbons may have contributed to the chemical evolution of the atmosphere from prior to the origin of life until today.

## Introduction

Methane (CH_4_) is a potent greenhouse gas which has in the past and is still today contributing to climate change^1^. Atmospherically accumulated CH_4_ and ethane (C_2_H_6_) might also explain the “faint young sun paradox”, which describes the apparent contradiction of a fainter sun (70 – 83% of the current solar energy output) but a climate that was at least as warm as today during early Earth (4.5–2.5 Ga ago)^2–4^. Although these CH_4_ levels would be essential to keep the Earth a liquid hydrosphere to allow the evolution of life during the Archean (4.0–2.5 Ga), the source of CH_4_ prior to the origin of life is still under debate^5^. While CH_4_ was released by submarine volcanism, most CH_4_ is suggested to be formed as side product of serpentinization^5^. After the evolution of microbial methanogenesis latest by 3.5 Ga^6^, methanogenesis could have been responsible for a CH_4_ flux comparable to today^7^. Thus, methanogenesis is expected to be the main source of CH_4_ during the Archean, supported by light carbon isotope values in sedimentary deposits^8^. However, isotope signals can only manifest upon reoxidation and CH_4_ itself does not leave much of a signature in the geological record. Thus, the actual CH_4_ concentrations and the potential abiotic sources during early Earth remain elusive. Based on mass-independent fractionation of sulfur, at least 20 ppmv CH_4_ was present around 2.4 Ga ago^9^. A more recent study analyzing the fractionation of xenon isotopes suggests CH_4_ levels of >5000 ppmv around 3.5 Ga ago^10^. Catling et al. expect even higher CH_4_ levels at the beginning of the Archean (4 Ga)^3^ before methanogenesis evolved. Yet, the processes responsible for these high CH_4_ levels and their relative contributions remain controversial.

Recently, we discovered a non-enzymatic CH_4_ formation mechanism expected to occur in all living organisms^11^. The mechanism has been demonstrated to be active in over 30 very diverse organisms^11^ and suggested to explain previously observed CH_4_ formation by cyanobacteria^12^, freshwater and marine algae^13,14^, saprotrophic fungi^15^ and plants^16^. The CH_4_ formation is driven by a cascade of radical reactions, governed by the interplay of reactive oxygen species (ROS) and ferrous iron (Fe^2+^), methylated sulfur (S)- and nitrogen (N)-compounds are oxidatively demethylated by hydroxyl radicals (∙OH) and oxo-iron(IV) complexes ([Fe^IV^=O]^2+^) to yield methyl radicals (·CH_3_)^11^.

**Fig. 1:**
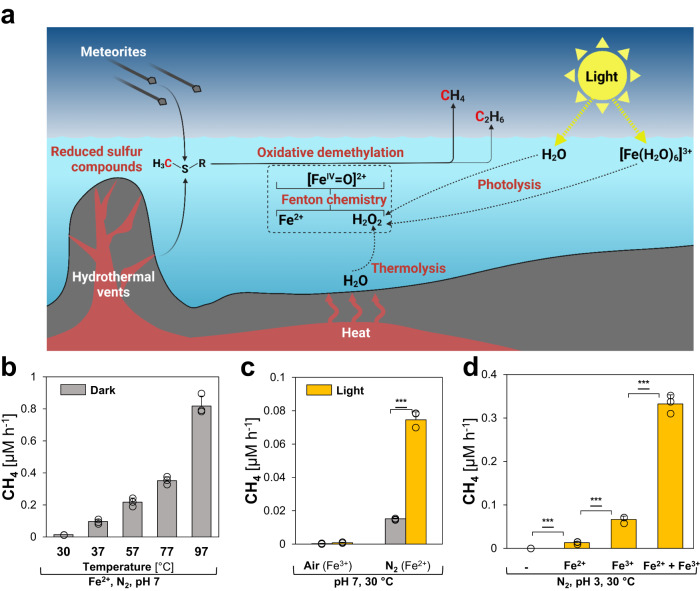
Heat and light drive CH_4_ formation under abiotic conditions. **a** Reduced, methylated S-/N-compounds are formed abiotically in hydrothermal vents or transported to Earth by carbonaceous meteorites. Under anoxic conditions, H_2_O_2_ is formed by thermolysis and photolysis of water and [Fe(H_2_O)_6_]^3+^ complexes, reacting with dissolved ferrous iron (Fe^2+^) to hydroxyl radicals (∙OH) and [Fe^IV^= O]^2+^ compounds that drive the oxidative demethylation of methylated S-/N-compounds, thereby facilitating CH_4_ and C_2_H_6_ formation. **b** Thermolysis: CH_4_ is formed from DMSO under high temperatures. **c** Water photolysis: The formation of CH_4_ is increased by light. **d** [Fe(H_2_O)_6_]^3+^ photolysis: Under acidic conditions, light-driven CH_4_ formation is enhanced by [Fe(H_2_O)_6_]^3+^ photochemistry. All experiments were conducted in closed glass vials containing buffered solutions (pH 7 or pH 3) supplemented with DMSO and Fe^2+^ or Fe^3+^ at 30 °C (**b**, **c**) under a N_2_ or air atmosphere. Statistical analysis was performed using paired two-tailed *t* tests, ***p ≤ 0.001. The bars are the mean + standard deviation of triplicates, shown as circles. **a** Was created with BioRender.com.

Here we show that this abiotic mechanism occurs also outside living cells and might have contributed to CH_4_ levels before life emerged. All needed components: (i) methylated S- and N-compounds, (ii) Fe^2+^ and (iii) ROS are found under early-earth conditions. (i) In a prebiotic world, methylated S-compounds like methanethiol, dimethyl sulfide (DMS) or dimethyl sulfoxide (DMSO) were formed abiotically under the reducing conditions of hydrothermal vents^17–19^ or transported to Earth by carbonaceous meteorites during early Earth meteorite bombardment^20,21^. Upon the emergence of life, more methylated S-/N-compounds were produced by cells and organisms, i.e. methionine, dimethylsulfoniopropionate or trimethylamine^22^. (ii) Under the anoxic conditions of the early Earth, oceans were rather ferruginous, i.e. rich in Fe^2+^ required for Fenton chemistry^23,24^, nonetheless ferric iron (Fe^3+^) also occurred in Archean seawater^25^. Additionally, the mechanism driven by Fe^2+^ can be enhanced by Fenton-promoting Fe^2+^-chelators, e.g. ATP or citrate^26^. Under anoxic conditions, Fe(III)-carboxylate complexes are photochemically reduced via ligand-to-metal charge transfer (LMCT)^27^, resulting in Fe^2+^ and organic radicals^28^. (iii) Under ambient temperatures, low ROS levels exist in water that increase with heat^29^, or can be generated by photolysis or radiolysis^30–33^. Under acidic conditions, i.e. in volcanic lakes^34^, illumination of Fe(III)-aqua complexes ([Fe(H_2_O)_6_]^3+^) forms Fe^2+^ and ROS^35,36^. Thus, we hypothesized that the Fenton reaction of Fe^2+^ with H_2_O_2_, generated by heat and light, could have driven the formation of CH_4_ from methylated S-/N-compounds independent of temperatures and pressures occurring at hydrothermal vents but at ambient conditions as early as the prebiotic world of the Hadean (4.5–4.0 Ga, Fig. 1a). To identify critical components of such a mechanism, we used aqueous model systems to determine the influence of heat, light, and (bio)molecules on CH_4_ formation in abiotic and biotic environments.

## Results

### Methane is formed under abiotic conditions

To investigate CH_4_ formation under abiotic conditions (Fig. 1a), we designed a chemical model system consisting of a nitrogen atmosphere, a potassium phosphate-buffered solution (pH 7, expected during the Archean at 4.0 Ga^37^) supplemented with Fe^2+^ and the abiotically formed DMSO which serves as methyl donor for ROS-driven CH_4_ formation. Over the course of the experiments, no pH change was observed, while low amounts of Fe(OH)_2_ precipitated. In this model system, CH_4_ was consistently formed from DMSO in the dark (Fig. 1b). CH_4_ formation rates increased with rising temperatures from 30 to 97 °C, consistent with the previously reported temperature-dependency of ROS levels in water^29^. While only marginal CH_4_ formation rates derived from DMSO were observed at 30 °C (~0.02 μM h^−1^), rates increased 41-fold to ~0.82 μM h^−1^ at 97 °C. In addition, low C_2_H_6_ amounts were formed (Supplementary Fig. 1), most likely resulting from the recombination of two methyl radicals^23^. At 37 °C, the CH_4_:C_2_H_6_ ratio was ~110, with an increasing trend towards higher temperatures. As the ROS-driven CH_4_:C_2_H_6_ ratios are substantially lower than those observed for archaeal methanogenesis^38^, the CH_4_:C_2_H_6_ ratios could serve as indicator to distinguish microbial from abiotic processes.

Light enhanced the abiotic CH_4_ formation rates (Fig. 1c) by photolysis of water and generation of H_2_O_2_ at 30 °C (Supplementary Fig. 2). Notably, CH_4_ amounts increased ~4-fold from ~0.02 μM h^−1^ to ~0.08 μM h^−1^ upon broad-spectrum illumination (~ 350 nm < λ < ~1010 nm at 82 ± 4 µmol photons m^−2^s^−1^, Supplementary Fig. 3). This data provides evidence that light-driven CH_4_ formation from methylated S-compounds can occur even in the absence of biomolecules. The addition of oxygen to the samples stopped the formation of CH_4_ in this pH-neutral model system supplemented with Fe^3+^ (Fig. 1c). In contrast, under acidic (pH 3), illuminated conditions CH_4_ formation rates increased ~5-fold upon Fe^3+^-supplementation in comparison to Fe^2+^-addition, indicating light-driven ROS and Fe^2+^ formation from [Fe(H_2_O)_6_]^3+^ complexes (Fig. 1d)^35^. Upon supplementation of 1 mM Fe^3+^ and 1 mM Fe^2+^, keeping the overall iron concentration unchanged at 2 mM, CH_4_ formation rates increased to ~0.33 μM h^−1^. This 5-fold rate increase is driven by both ROS-inducing Fe^3+^ and Fenton-driving Fe^2+^. Under pH-neutral conditions, mixing Fe^2+^ and Fe^3+^ only increased CH_4_ formation rates by ~1.3-fold in comparison to Fe^2+^-supplemented samples, while only trace amounts of CH_4_ were obtained from Fe^3+^-supplemented samples (Supplementary Fig. 4). Thus, illuminated [Fe(H_2_O)_6_]^3+^ complexes generate both Fe^2+^ and ROS, thereby contributing to the ROS-driven CH_4_ formation under acidic conditions.

Taken together, we demonstrated that heat and light drive the formation of CH_4_ and C_2_H_6_ in an anoxic, abiotic environment under ambient temperatures and pressures. These results establish a ROS-driven mechanism based on Fenton chemistry that can occur delocalized from serpentinization across Earth’s humid realm and thereby substantially differs from previously suggested mechanisms that are spatially restricted. Thus, this non-enzymatic hydrocarbon formation mechanism could have released CH_4_ and C_2_H_6_ into the atmosphere of the Hadean and Archean. Besides CH_4_, C_2_H_6_ is considered an important factor in keeping the early Earth warm, since C_2_H_6_ absorbs from 11 to 13 µm in an atmospheric window (roughly 8–13 µm) where H_2_O and CO_2_ do not absorb strongly^2^. Together, the hydrocarbons produced by these pathways might offer a solution to the “faint young sun paradox”^3,4^.

### (Bio)molecules enhance the heat-driven CH_4_ formation

**Fig. 2:**
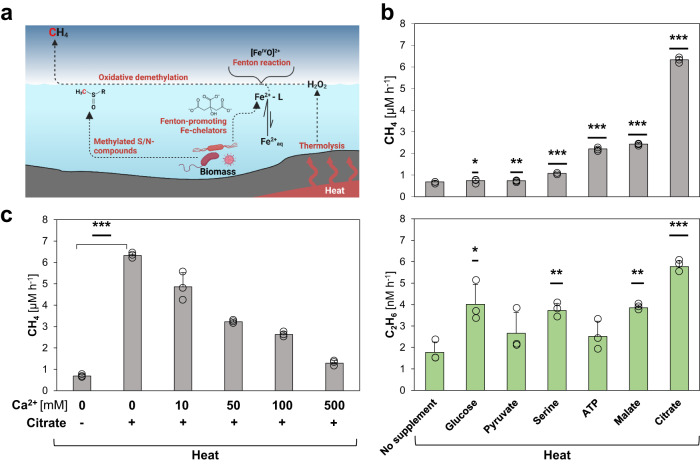
(Bio)molecules enhance heat-driven CH_4_ formation. **a** Overview of CH_4_ formation driven by heat. Living organisms produce S-/N-methylated compounds that serve as substrates for CH_4_ formation and Fe^2+^-chelators that promote Fenton chemistry and enhance CH_4_ formation. **b** Heat-driven CH_4_ (upper panel) and C_2_H_6_ (lower panel) formation is enhanced upon supplementation with (bio)molecules. **c** Citrate enhances heat-driven CH_4_ formation acting as iron-chelator. Upon the addition of Ca^2+^, CH_4_ levels decrease due to the replacement of Fenton-promoting Fe^2+^-citrate complexes with Ca^2+^-citrate complexes. All experiments were conducted in closed glass vials containing a buffered solution (pH 7) supplemented with DMSO, Fe^2+^ and, optionally, citrate and Ca^2+^ under a pure nitrogen atmosphere at 97 °C (heat). Statistical analysis was performed using paired two-tailed *t* tests, *p ≤ 0.05, **p ≤ 0.01, ***p ≤ 0.001. The bars are the mean + standard deviation of triplicates, shown as circles. **a** Was created with BioRender.com.

Even before life emerged, several metabolites, e.g. citrate and malate, could have been formed via an ancient, non-enzymatic TCA cycle predecessor driven by ROS^39,40^. Catalyzed by iron particles, the formation of pyruvate from CO_2_ was recently reported^41^. Intriguingly, citrate and malate, as well as other primordial (bio)molecules with a putative prebiotic origin, including ATP^42^ or serine^43^, have been reported to act as Fenton-promoting Fe^2+^-chelators^26^. We therefore investigated if these hydroxylated and carboxylated (bio)molecules enhance the ROS-driven CH_4_ formation rates (Fig. 2a).

Indeed, the addition of pyruvate, glucose, serine, ATP, malate or citrate to the heat-driven (97 °C) model system increased the abiotic CH_4_ formation rate, e.g. more than 11-fold for citrate (Fig. 2b). Corresponding C_2_H_6_ rates significantly increased for glucose, serine, malate and citrate, resulting in CH_4_:C_2_H_6_ ratios between ~190 (glucose) and ~1100 (citrate, Fig. 2b). To test if these enhancing effects were indeed driven by Fe^2+^ chelation, we supplemented the assays with the Fe^2+^-competitor Ca^2+^ (Fig. 2c). Since (bio)molecules like citrate can alternatively chelate Ca^2+^ ions, we expected that increasing Ca^2+^ concentrations result in decreasing CH_4_ formation rates by replacing Fenton-promoting Fe^2+^-citrate complexes with Ca^2+^-citrate complexes. Upon addition of 10 mM and 500 mM Ca^2+^, CH_4_ formation rates significantly decreased from ~6.32 μM h^−1^ to ~4.86 μM h^−1^ and ~1.29 μM h^−1^, respectively. Thus, 500 mM Ca^2+^ suppressed ~90% of the Fenton-promoting effect of citrate supplementation. The Ca^2+^ concentration-dependent decrease of the heat-driven CH_4_ formation rate supports the role of citrate as a Fenton-promoting Fe^2+^-chelator, which is further indicated by citrate dissolving any ferruginous precipitate.

Together, ROS generated by heat interact with iron and thereby drive the formation of methyl radicals from S-/N-methylated compounds, resulting in CH_4_ and C_2_H_6_. Moreover, several hydroxylated or carboxylated (bio)molecules with a putative prebiotic origin were shown to act as Fenton-promoting Fe^2+^-chelators, indicating that ROS-driven CH_4_ formation may have already been widespread within the timeframe of the transition from prebiotic chemistry to the origin of life. The rise of life would have fostered the abiotic, non-enzymatic CH_4_ formation due to the consequential formation and release of biomolecules serving as chelators and substrates.

### A light-driven iron redox cycle sustains CH_4_ formation

**Fig. 3:**
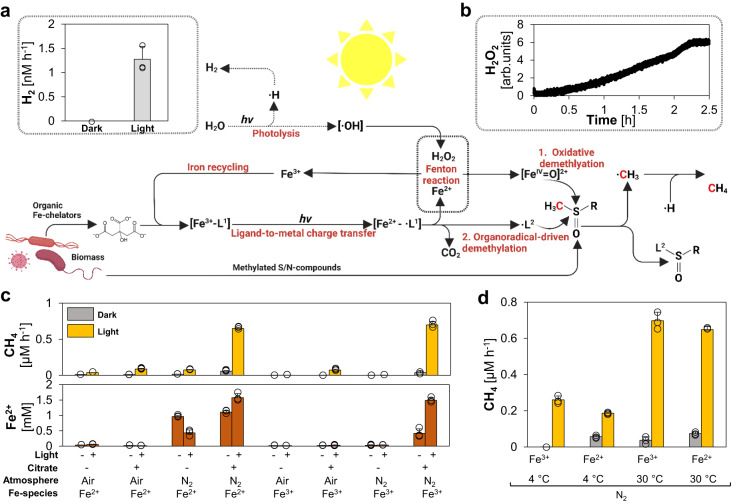
A light-driven iron redox cycle drives and enhances CH_4_ formation. Upon illumination, water is photolytically split into hydroxyl radicals (·OH) and hydrogen forming H_2_ and H_2_O_2_. Organic Fe^3+^-complexes (Fe^3+^-[L^1^]) are converted into Fe^2+^ and organic radicals (·L^1^) via ligand-to-metal charge transfer (LMCT). The generated Fe^2+^ reacts with H_2_O_2_ to ·OH or [Fe^IV^= O]^2+^ and thereby drives the generation of methyl radicals (·CH_3_) from S-/N-methylated compounds. The LMCT-generated ·L^1^ decomposes into CO_2_ and another organic radical (·L^2^) that additionally facilitates CH_4_ formation upon reacting with S-/N-methylated compounds. Under light, (**a**) H_2_ (gray bars) and (**b**) H_2_O_2_ is formed in pure buffer. **c** Upon illumination, CH_4_ formation rates (yellow bars) are increased. Fe^2+^ formation (brown bars) depends on anoxic conditions and is driven by LMCT induced by the addition of citrate. **d** Light and heat have synergistic effects on CH_4_ formation. While heat drives CH_4_ formation upon Fe^2+^-supplementation, light increases CH_4_ formation upon Fe^3+^- and Fe^2+^-addition. All experiments were conducted in closed glass vials containing a buffered solution (pH 7) supplemented with DMSO, Fe^2+^ or Fe^3+^, N_2_ or air atmosphere in the presence or absence of citrate incubated under light or in the dark at 4 °C or 30 °C. The bars are the mean + standard deviation of triplicates, shown as circles. **a**, **b** Was created with BioRender.com.

During Fenton chemistry, Fe^2+^ is either oxidized to [Fe^IV^= O]^2+^ or ferric iron (Fe^3+^). As Fe^3+^ cannot drive Fenton reactions^23,24^, CH_4_ formation rates decrease with increasing reaction time and increasing concentrations of Fe^3+^. While this effect may have been minor in the ferruginous Archean oceans, Fe^3+^ likely dominated the iron pool in the photic zone of the oceans latest by the rise of photoferrotrophy and was also prevalent in several ecological niches, e.g. volcanic lakes^34^. The evolution of photosynthesis and the subsequent biological production of O_2_ oxidized the majority of the available Fe^2+^ to Fe^3+^. Thus, abiotic ROS-driven CH_4_ formation would have been hindered in the sunlit realm by the late Archean in the absence of an iron redox cycle at neutral pH. Intriguingly, besides acting as Fenton-promoting Fe^2+^-chelators^26^, (bio)molecules like citrate were reported to reduce Fe^3+^ to Fe^2+^ via LMCT under oxic and anoxic conditions^27^. Therefore, (bio)molecules may have facilitated widespread iron redox cycling, e.g. by forming Fe(III)-carboxylate complexes. Furthermore, previous studies showed that, upon illumination of water hydroxyl radicals (·OH) and hydrogen atoms are generated, forming H_2_O_2_ and H_2_^30–33^. Thus, we hypothesized that light could drive CH_4_ formation in the absence of Fe^2+^ by simultaneously (i) generating ROS from water and (ii) reducing Fe^3+^ to Fe^2+^ via LMCT, thereby recycling Fe^3+^ and keeping the Fenton reaction running (Fig. 3).

To verify our hypothesis, we first confirmed light-dependent ROS production in our model system in the absence of substrate, iron and organic ligands by measuring final reaction products of photolysis: H_2_ and H_2_O_2_ (Fig. 3a, b). We measured H_2_ production at a rate of ~1.3 nM h^−1^ in anoxic samples under broad-spectrum illumination but not in samples kept in the dark (Fig. 3a). A continuous formation of H_2_O_2_ was measured online using microsensors, which confirmed light-dependent production dynamics in pure buffer (Fig. 3b). Via fluorescence-based H_2_O_2_ endpoint measurements, we found that both iron and DMSO reduced the H_2_O_2_ concentrations. The decrease in H_2_O_2_ levels can be attributed to Fenton reactions between H_2_O_2_, Fe^2+^ and the radical scavenger DMSO (Supplementary Fig. 5).

Building on this, we closely investigated the interplay of LMCT and iron photochemistry on CH_4_ formation. For this purpose, we analyzed our chemical model system containing a buffered solution (pH 7), Fe^2+^ or Fe^3+^, DMSO, in the presence or absence of citrate for the formation of CH_4_ and the concentration of available Fe^2+^ (Fig. 3c). The influence of the following parameters on the formation of CH_4_ was tested: (i) O_2_ ( ~ 21% in air), (ii) oxidation state of the supplemented iron species (Fe^2+^ *vs*. Fe^3^), (iii) light and (iv) presence/absence of citrate. (i) CH_4_ formation rates under anoxic conditions always exceeded rates under oxic conditions. (ii) Without citrate, initial Fe^2+^-supplementation was required to form significant CH_4_ levels. (iii) CH_4_ formation always increased with light. (iv) Upon citrate addition, CH_4_ formation was enhanced in illuminated and anoxic samples containing DMSO and Fe^2+^ or Fe^3+^. Besides elevated CH_4_ formation rates, citrate addition also increased the final Fe^2+^ concentrations, e.g. from ~0 mM Fe^2+^ to ~1.5 mM Fe^2+^ in illuminated and anoxic samples.

After determining the influence of the four parameters (i) O_2_, (ii) iron (iii) light and (iv) (bio)molecules, we further investigated them individually to gain a better understanding of their contribution and role in the light-driven CH_4_ formation.

**Fig. 4:**
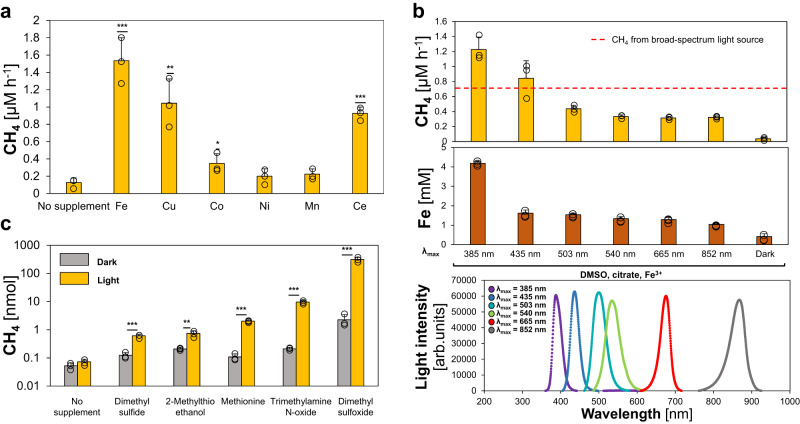
Transition metals, wavelengths and methylated sulfur- and nitrogen compounds mediate light-driven CH_4_ formation. **a** Iron, cobalt and cerium enhance light-driven CH_4_ formation. No significant CH_4_ increase was observed for cobalt, nickel and manganese supplementation. **b** Light-driven CH_4_ formation and Fe^2+^ generation increases in the near-UV spectrum. **c** Light-driven formation of CH_4_ from methylated S-/N-compounds (logarithmic scale). Upon illumination, significant increases in CH_4_ levels were measured for dimethyl sulfide, methionine, 2-methylthioethanol, trimethylamine N-oxide and dimethyl sulfoxide (DMSO). All experiments were conducted in closed glass vials containing a buffered solution (pH 7), N_2_ and either Fe^3+^ or other transition metals (**a**), DMSO or other substrates (**c**) and either ascorbate (**a**) or citrate (**b**, **c**). Samples were incubated under broad-spectrum light (**a**, **c**), specific wavelengths (**b**) or in the dark at 30 °C. The dashed red line depicts the average CH_4_ amounts obtained from samples illuminated by a broad-spectrum light source. Statistical analysis was performed using paired two-tailed *t* tests, *p ≤ 0.05, **p ≤ 0.01, ***p ≤ 0.001. The bars are the mean + standard deviation of triplicates, shown as circles.

(i) O_2_: The influence of O_2_ on LMCT and CH_4_ formation was studied in citrate-supplemented samples by adding various amounts of air. Fe^2+^ concentrations and CH_4_ formation rates decreased with increasing O_2_ levels (Supplementary Fig. 6). In comparison to 0 % O_2_, the Fe^2+^ concentration dropped drastically already at 0.2 % O_2_ and was ~96 % lower at 2 % O_2_, while CH_4_ formation rates decreased approximately linearly with the O_2_ level. This indicates the presence of a Fe-cycle, in which most LMCT-formed Fe^2+^ is instantly re-oxidized, either by O_2_ or Fenton reactions. The balance between these Fe^2+^ sinks depend on O_2_ availability and governs CH_4_ formation rates. In the presence of O_2_, we also detected methanol (CH_3_OH) formation rates ranging from ~0.003 μM h^−1^ (0.2 % O_2_) to ~0.07 μM h^−1^ (21 % O_2_). CH_3_OH is preferentially formed through the reaction of ·CH_3_ with O_2_^23,44^. Without the addition of O_2_, no CH_3_OH was detected, indicating anoxic conditions in our standard assays.

(ii) Iron: The role of the LMCT-rate and the corresponding Fe^2+^ availability for CH_4_ formation was tested by supplementing the assays with various Fe^3+^ concentrations (Supplementary Fig. 7). At lower Fe^3+^ concentrations, CH_4_ formation rates increased steeper than the measured Fe^2+^ concentrations. At high Fe^3+^ concentrations, CH_4_ formation rates leveled off, while Fe^2+^ concentrations continued to increase. This indicates that Fe^2+^ is limiting the demethylation rates at low iron concentrations, because it is immediately re-oxidized, while light-dependent ROS production is limiting CH_4_ formation at high iron concentrations. Most importantly, these data highlight that a light- and ROS-driven iron cycle can facilitate high rates of CH_4_ formation, even in the presence of O_2_ and the absence of detectable Fe^2+^, which opens the possibility of widespread abiotic CH_4_ production after the great oxidation event as well as in diverse modern habitats. Next, we investigated the role of the alkali metal magnesium (Mg^2+^) due to its high environmental abundance and found that Mg^2+^ does not facilitate CH_4_ formation in illuminated buffer containing DMSO and citrate (Supplementary Fig. 8). Upon additional Fe^3+^ supplementation, Mg^2+^ also decreased CH_4_ formation rates by replacing Fenton-promoting Fe^3+^-citrate complexes by Mg^2+^-citrate complexes, thereby acting similar to Ca^2+^ that was demonstrated to decrease heat-driven CH_4_ formation (Fig. 2c). Besides iron, the transition metals copper, cerium, cobalt, nickel and manganese were reported to drive Fenton chemistry^45,46^, resulting in the release of CH_4_. Thus, we tested different transition metals in our chemical model system, containing DMSO as substrate and ascorbate as a strong metal reductant^47,48^. We observed that copper, cobalt and cerium also enhanced CH_4_ formation rates (Fig. 4a). However, the activity of copper, cobalt and cerium was lower than iron. The high activity of iron combined with its ubiquitous abundance in the Precambrian highlights the global distribution and importance of this mechanism.

(iii) Light: It is established that light quality has an important influence on photolysis. Short wavelength light in the ultraviolet spectrum was reported to drive water photolysis and LMCT more efficiently than longer wavelengths^49^. We expected that shorter wavelength light would increase both CH_4_ formation rates and Fe^2+^ levels. Indeed, CH_4_ formation rates surged from ~0.3 μM h^−1^ (λ_max_ = 534 nm) to ~1.23 μM h^−1^ (λ_max_ = 388 nm, Fig. 4b) and Fe^2+^ concentrations almost tripled from ~1.3 mM (λ_max_ = 534 nm) to ~4.2 mM (λ_max_ = 388 nm). Although the broad-spectrum light had a 1.5-fold higher energy flux (57 ± 2 kJ m^−2^ h^−1^) compared to the 388 nm-LED light (37 ± 2 kJ m^−2^ h^−1^), the CH_4_ formation rate under the broad-spectrum light was only half (0.7 μM h^−1^). Given that the stratospheric ozone layer was absent during the Hadean and Archaean, higher fluxes of short wavelength light (*i.e*. ultraviolet light), reached aqueous environments and may have further enhanced the ROS-driven CH_4_ formation.

(iv) (Bio)molecules: After illumination of Fe^3+^-ligand complexes, one electron is transferred via LMCT from a carboxylated ligand (L^1^) to Fe^3+^, an organic radical (·L^1^), i.e. citrate radical, is generated. As described in the literature^28^, we observed the subsequent CO_2_ disassembly from citrate radicals (Supplementary Fig. 9). We speculated that the remaining organic radical (·L^2^) could react with DMSO, resulting in ·CH_3_ and the formation of CH_4_ (Fig. 3). Since we cannot directly detect organic radicals, we mimicked the proposed reaction in an anoxic model system only containing DMSO and the radical-generating 2,2’-azobis(2-amidinopropane) dihydrochloride (APPH) that readily decomposes into carbon-centered organic radicals at 40 °C (Supplementary Fig. 10). Indeed, we observed CH_4_ formation in a mixture of DMSO and APPH, while only trace amounts of CH_4_ were observed from either DMSO or AAPH alone, suggesting an organic radical-driven CH_4_ formation mechanism. In short, carboxylates like citrate facilitate LMCT, thereby reducing Fe^3+^ to Fe^2+^ and forming organic radicals. Both resulting compounds drive CH_4_ formation. Overall, CH_4_ can be formed under anoxic conditions via (i) water thermolysis, (ii) water photolysis, (iii) [Fe(H_2_O)_6_]^3+^ photolysis and (iv) LMCT-induced carbon-centered radicals. Apart from serving as chelators, some (bio)molecules could also serve as substrates for Fenton reactions. Thus, we investigated four S-/N-methylated compounds in the presence of the chelator citrate. Upon illumination, CH_4_ was formed from dimethyl sulfide, methionine, 2-methylthioethanol and trimethylamine (Fig. 4c). These observations indicate that ROS-driven CH_4_ formation significantly increased after the origin of life by providing biomolecules as chelators and substrates.

Finally, synergistic effects between light and heat were observed (Fig. 3d). For Fe^2+^-supplemented samples, CH_4_ rates at 4 °C increased from ~0.056 μM h^−1^ in the dark over ~0.19 μM h^−1^ under light to ~0.65 μM h^−1^ in illuminated samples at 30 °C. For Fe^3+^-supplemented samples, only CH_4_ rates below 0.03 μM h^−1^ were obtained in the dark, while CH_4_ formation rates were slightly above Fe^2+^-supplemented samples in the light, again demonstrating the effects of LMCT and LMCT-induced carbon-centered radicals. Thus, the two factors heat and light synergistically combine for a stable and enhanced ROS and CH_4_ formation.

### Biomass-derived CH_4_ with an abiotic isotope fractionation

Considering the impact of (bio)molecules on the LMCT-driven Fenton reaction, organic radical generation and the role of biomolecules as substrates, we expect the discussed mechanisms to have played and still play the most important role in the vicinity of decaying biomass. To demonstrate that CH_4_ is indeed formed from dead biomass in the presence of a variety of biomolecules and not just in our well-defined model systems, we conducted deuterium labeling experiments. For this purpose, we grew the bacterium *B. subtilis* in *Luria-Bertani* medium supplemented with 10% D_2_O and inactivated the cells by sonication and freezing (see Methods).

**Fig. 5:**
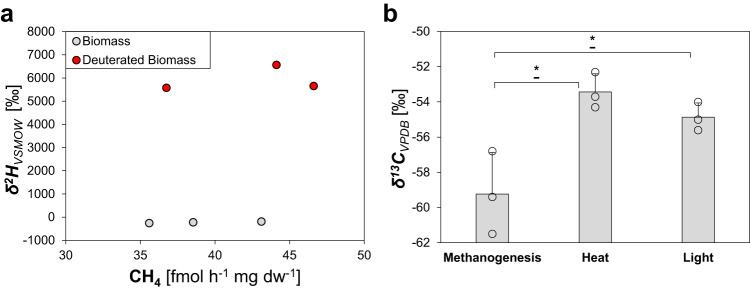
Isotope labeling studies confirm dead biomass as substrate and show an abiotic isotope fractionation for ROS-driven CH_4_ formation. **a** Unlabeled or deuterium-enriched CH_4_ is formed from unlabeled biomass (gray dots) or deuterated biomass (red dots), respectively. **b** Stable carbon isotope values of cultures from the methanogen *Methanothermobacter marburgensis*, heat-, or light-generated CH_4_. All experiments were conducted in closed glass vials containing a buffered solution (**a**, **b**—heat, light) or culture medium (**b**—methanogenesis), supplemented with Fe^3+^ and ascorbate (**a**) or Fe^2+^ and citrate (**b**—heat, light) under a nitrogen atmosphere, incubated under light at 30 °C or in the dark at 97 °C. Statistical analysis was performed using paired two-tailed *t* tests, *p ≤ 0.05. The bars are the mean + standard deviation of triplicates, shown as circles.

The obtained dead biomass was supplemented with Fe^3+^ and ascorbate and incubated under broad-spectrum light. Around 40 fmol CH_4_ h^−1^ mg^−1^ dry weight was obtained from labeled and unlabeled biomass (Fig. 5a). In addition, stable hydrogen isotope values (*δ*^*2*^*H)* of CH_4_ from D_2_O-treated biomass showed strong enrichment in deuterium (~5900 ‰) in comparison to unlabeled biomass (~−225 ‰), demonstrating a direct conversion of isotopically labeled biomass to CH_4_. This suggests that the availability of biomass, upon the emergence of life, has increased the CH_4_ formation by delivering both (i) S-/N-methylated compounds and (ii) Fenton-promoting iron chelators. The presence of CH_4_ has been suggested to be crucial for the evolution of life, since it could serve as life´s first carbon source via methanotrophy^50–52^. Following this line of thought, we could demonstrate that methanotrophic *Methylocystis hirsuta* grew on CH_4_ generated by our light-driven model system, transferred to the headspace of the *M. hirsuta* culture (Supplementary Fig. 11). In fact, the “last methane-metabolizing ancestor” had likely the genes to perform methanogenesis and anaerobic methane oxidation^53^, suggesting that, under high CH_4_ concentrations, methanotrophy could have emerged prior to methanogenesis.

Finally, we speculated that ROS-driven CH_4_ formation leads to different stable carbon isotope values (*δ*^*13*^*C*) compared to biological processes, *i.e*., methanogenesis. The observed *δ*^*13*^*C* values for CH_4_ generated by heat or light were less negative (~−54 ± 1.1‰) compared to the *δ*^*13*^*C* value of the methanogen *Methanothermobacter marburgensis* (~−59.2 ± 2.3‰, Fig. 5b). While the isotopic fractionation during abiotic ROS-driven CH_4_ formation remains to be studied in depth, these results suggest a lower carbon isotope fractionation for ROS-driven CH_4_ formation than for enzymatic methanogenesis. Together with the observed CH_4_:C_2_H_6_ ratios, isotopic signatures may therefore serve to differentiate between CH_4_ formed enzymatically or abiotically on Earth and extraterrestrial planets.

## Discussion

In this work, we demonstrated that the interplay of Fe^2+^ and H_2_O_2_, generated by heat and light, drives CH_4_ and C_2_H_6_ formation from methylated S-/N-compounds via Fenton chemistry under conditions that were globally prevalent in the Hadean and Archean. As we observed CH_4_ formation under suboxic and oxic conditions, these mechanisms could, in principle, also contribute to extant CH_4_ emissions from aqueous environments that were recently shown to correlate with light instead of specific enzymatic pathways^54^. The here described pathways allow CH_4_ and C_2_H_6_ formation in many aqueous environments including oceans, lakes, rivers, and ponds, delocalized from restricted hotspots for (bio)molecule formation such as hydrothermal vents or ultramafic rocks, in superficial water layers driven by light and throughout the entire water column driven by heat. After the emergence of life, this phenomenon would have greatly intensified in the anoxic Archaean and the subsequent “boring billion”^55,56^. The increasing amounts of biomass provided methylated S-/N-substrates, Fe-chelating biomolecules reducing Fe^3+^ to Fe^2+^ and releasing organic radicals and thus enhance ROS-driven CH_4_ formation. Possibly, these reactions facilitated elevated CH_4_ and C_2_H_6_ levels during the Hadean and Archean. These hydrocarbons would have contributed to atmospheric temperatures on Earth and allowed the evolution of life in a liquid hydrosphere which could have influenced the evolution of metabolism by allowing the rise of methanotrophy prior to methanogenesis. This work lays the foundation to explore further the mechanism’s role in shaping the evolution of the atmosphere on Earth and other planets and its influence on the current climate change.

## Methods

### General assay conditions

Unless otherwise indicated, 4 mL samples were incubated in closed 20 mL glass vials at 30 °C under a pure nitrogen (N_2_) atmosphere and subsequently analyzed via gas chromatography (GC).

### Heat assays

In total, 500 mM DMSO and 10 mM FeSO_4_ were added to 20 mM degassed potassium phosphate buffer (pH 7) in an anaerobic tent. The headspace of the closed vials was then cycled three times with vacuum and N_2_. Samples were incubated at 37 °C, 57 °C, 77 °C and 97 °C for 6 h in an incubator in the dark. Optionally, 20 mM citrate, malate, ATP, serine, glucose or pyruvate were also supplemented. Ca^2+^ was added in the form of CaCl_2_. Samples were measured within the linear range of CH_4_ formation rate via gas chromatography.

### Light assays

In total, 500 mM DMSO, 2 mM of either FeCl_3_ or FeSO_4_ and, optionally, 10 mM citrate were added to 20 mM degassed potassium phosphate buffer (pH 7). Anoxic conditions were generated by drawing vacuum eight times for 1 min and a subsequent filling with N_2_. For experiments investigating [Fe(H_2_O)_6_]^3+^ complexes, samples were incubated under anoxic, acidic conditions (20 mM Tris · HCl buffer, pH 3) and supplemented with 500 mM DMSO and either 2 mM FeCl_3_, 2 mM FeSO_4_ or 1 mM FeCl_3_ and 1 mM FeSO_4_, each. For the investigation of transition metals (Fig. 4a), 2 mM cerium (CeNH_4_SO_4_), manganese (MnSO_4_), cobalt (CoNO_3_), nickel (NiSO_4_), copper (CuCl_2_) or iron (FeCl_3_) and 10 mM pH-neutral ascorbate were added to 500 mM DMSO and 20 mM potassium phosphate buffer with an incubation for 1 day. The effect of different wavelengths on CH_4_ formation (Fig. 4b) was investigated by adding 5 mM FeCl_3_, 10 mM citrate and 500 mM DMSO to 20 mM potassium phosphate buffer with an incubation for 1 day. For the determination of substrates for CH_4_ formation (Fig. 4c), 500 mM DMS, methionine, 2-methylthioethanol, Trimethylamine N-oxide or DMSO were added to 10 mM FeCl_3_ and 100 mM citrate in 20 mM potassium phosphate buffer with an incubation for 3 days. Samples were incubated under air or N_2_ in the dark or under constant broad-spectrum illumination from light bulbs (Osram, Superlux, Super E SIL 60; Φ = 82 ± 4 µmol photons m^−2^ s^−1^, *H* = 52 ± 2 kJ m^−2^ h^−1^; Supplementary Fig. 3) for 1 day. Samples were measured within the linear range of the CH_4_ formation via gas chromatography. Specific wavelengths were provided by diodes (H2A1 series, Roithner Lasertechnik, Austria) emitting UV-A, blue, cyan, green, red or near-infrared light (λ_max_ = 388 nm, Φ = 35 ± 1 µmol photons m^−2^ s^−1^, *H* = 36 ± 2 kJ m^−2^ h^−1^; λ_max_ = 436 nm, Φ = 45 ± 1 µmol photons m^−2^ s^−1^, *H* = 45 ± 1 kJ m^−2^ h^−1^; λ_max_ = 500 nm, Φ = 64 ± 4 µmol photons m^−2^ s^−1^, *H* = 55 ± 3 kJ m^−2^ h^−1^; λ_max_ = 534 nm, Φ = 63 ± 1 µmol photons m^−2^ s^−1^, *H* = 50 ± 1 kJ m^−2^ h^−1^; λ_max_ = 675 nm, Φ = 45 ± 4 µmol photons m^−2^ s^−1^, *H* = 29 ± 3 kJ m^−2^ h^−1^; or λ_max_ = 868 nm, Φ = 69 ± 7 µmol photons m^−2^ s^−1^, *H* = 35 ± 3 kJ m^−2^ h^−1^) Light intensity was determined using a fiber optic scalar irradiance microsensor^57^ connected to a spectrometer (USB4000; Ocean Optics, USA) placed in the center of the incubation vials and calibrated using a spherical light probe (Walz) connected to a LI-250A light meter (Li-Cor Biosciences GmbH, Germany)^58^. Concentration of Fe^2+^ was quantified with the colorimetric ferrozine method^59^.

### Bacillus subtilis biomass assays

*B. subtilis* was grown in 500 mL LB media, supplemented with 10 % H_2_O or D_2_O, grown for 36 h at 37 °C and 180 rpm. The obtained culture was collected by three cycles of centrifugation (10 min, 4743 × *g*) and resuspended in 35 mL 20 mM potassium phosphate buffer (pH 7) in order to remove the excess D_2_O. Biomass was then generated by sonication (4-times, 1 min) and freezing of the samples. Subsequently, 80 mL buffer was supplemented with 10 mL biomass, 20 mM FeCl_3_ and 50 mM ascorbic acid, saturated with N_2_ for 30 min and incubated in 100 mL closed glass vials under N_2_ and constant broad-spectrum illumination for 3 days. The gas headspace was extracted with a syringe and analyzed with regard to CH_4_ content and *δ*^*2*^*H* values.

### Methylocystis hirsuta and Methanothermobacter marburgensis cultivation

*M. hirsuta* growth media contained 0.5 g Na_2_HPO_4_ · 2H_2_O, 0.22 g KH_2_PO_4_, 1 g KNO_3_, 0.4 mg CaCl_2_ · 2H_2_O, 2 mg MgSO_4_ · 7H_2_O per liter, supplemented with 5 mg Na_2_EDTA, 0.06 mg CuCl_2_ · 5H_2_O, 2 mg FeSO_4_ · 7H_2_O, 0.1 mg ZnSO_4_ · 7H_2_O, 0.03 mg MnCl_4_ · 4H_2_O, 0.05 mg H_3_BO_3_, 0.2 mg CoCl_2_ · 6H_2_O, 0.02 mg NiCl_2_ · 6H_2_O and 0.03 mg Na_2_MoO_4_ · 2H_2_O per liter. *M. hirsuta* was cultivated in 100 mL closed glass vials containing 30 mL culture and was incubated at 25 °C and 150 rpm under an air atmosphere. Methane was produced by supplementing 2 L degassed 20 mM potassium phosphate buffer with 1 M DMSO, 25 mM FeSO_4_ and 50 mM ascorbic acid, incubating the solution under constant illumination in 1 L flasks and collecting the formed CH_4_ with syringes. *M. hirsuta* cultures were either supplemented with 25 mL light-generated CH_4_ or 25 mL pure N_2_. *M. marburgensis* was cultivated as previously described^60^.

### Continuous H_2_O_2_ measurements using microsensors

To visualize H_2_O_2_ production in the illuminated anoxic model system, an H_2_O_2_ microsensor was positioned in the solution. The H_2_O_2_ microsensors were built, calibrated and used as described previously^61^. We sealed the vial opening with self-adhesive tape, rigorously bubbled the liquid with N_2_ and then adjusted a gentle flow of N_2_ through the headspace to minimize oxygen input from the atmosphere. Light was provided from halogen lamps (KL2500, Schott) at an intensity of 1027 µmol photons m^−2^ s^−1^. We did not attempt to calculate light-dependent H_2_O_2_ production rates due to the open design of the system, which allowed for the exchange of H_2_O_2_ with the headspace across the water interface.

### End-point H_2_O_2_ measurements

After illumination, 290 µL sample was mixed anaerobically with 9 µL Amplex Ultrared (Thermofisher, A36006, 30 µM final concentration) and 1 µL recombinant APEX2 (0.23 µM final concentration). Fluorescence was then measured with a plate reader (BMG ClarioStar™) at 568 nm excitation / 581 nm emission. A calibration curve was established with H_2_O_2_ following the same procedure. To prevent O_2_-driven H_2_O_2_ generation while sample preparation, all buffers were saturated with N_2_ and the plate reader was kept at a partial oxygen pressure of 0.1% with an atmospheric control unit (Clariostar, BMG). Before sample preparation, all sample components (20 mM potassium phosphate buffer, DMSO, 1 M citrate and 100 mM FeCl_3_) were degassed and kept in an anoxic tent overnight.

### Quantification of CH_4_, C_2_H_6,_ CO_2_, and H_2_ (GC-FID)

Amounts of formed CH_4_, C_2_H_6_, CO_2_ and H_2_ were determined via headspace analysis using a PerkinElmer® Clarus®690 GC system (GC–‍FID/TCD) with a custom-made column circuit (ARNL6743). The headspace samples were injected by a TurboMatrixX110 (PerkinElmer Inc, Waltham, USA) autosampler, heating the samples to 45 °C for 15 min prior to injection. The samples were then separated on a HayeSep column (7’ HayeSep N 1/8” Sf; PerkinElmer®), followed by molecular sieve (9’ Molecular Sieve 13×1/8” Sf; PerkinElmer®) kept at 60 °C. Subsequently, the gases were detected with a flame ionization detector (FID, at 250 °C) and a thermal conductivity detector (TCD, at 200 °C). The quantification of CH_4_, C_2_H_6_, CO_2_ and H_2_ was based on linear standard curves that were derived from measuring varying amounts of these gases.

### CH_3_OH measurements (GC-FID)

CH_3_OH was quantified with a GC-FID (Shimadzu GC-2010 Plus, FID-2010 Plus, 280 °C) containing an AOC 20i autosampler and a ZB-WAXplus (Zebron) column (30 m x ⌀ = 0.25 mm, df, 0.25 µm). A H_2_O sample (1 μL) was injected in the split liner (250 °C, split 5,15,50). The temperature program was kept at 35 °C for 5 min and then increased by 50 °C min^−1^ until 200 °C which was kept for 3 min. Helium served as carrier gas (flow rate: 1.95 ml min^−1^) and the FID was operated with 400 ml min^−1^ synthetic air, 40 ml min^−1^ H_2_ and 30 ml min^−1^ N_2_, serving as a makeup gas. For Split 5, a calibration curve (R^2^ = 0.9931) was generated by diluting CH_3_OH (99.9% purity), while an R^2^ = 0.9981 for split 15 and an R^2^ = 0.9997 for split 50 was determined.

### δ ^13^C stable isotope measurements (GC-C-IRMS)

δ^13^C values of CH_4_ were determined by gas chromatography-combustion-isotope ratio mass spectrometry (GC-C-IRMS). Aliquots of headspace gas were transferred to an evacuated sample loop (40 mL) and a cryogenic pre-concentration unit to trap CH_4_. CH_4_ was trapped on HayeSep D, separated from interfering compounds by GC and transferred to the GC-C-IRMS. The system consists of a cryogenic pre-concentration unit directly connected to an HP 6890 N GC (He flow rate: 1.8 mL min^−1^; Agilent Technologies, Santa Clara, USA) fitted with a GS-Carbonplot capillary column (30 m * 0.32 mm i.d., *d*_*f*_ 1.5 µm; Agilent Technologies) and a PoraPlot capillary column (25 m * 0.25 mm (i.d.), *d*_*f*_ 8 µm; Varian, Lake Forest, USA). The GC flow was coupled using a press-fit connector to a combustion reactor comprised of an oxidation reactor (ceramic tube (Al_2_O_3_), length 320 mm, inner diameter 0.5 mm, with oxygen-activated Cu/Ni/Pt wires inside; reactor temperature 960 °C) and a GC Combustion III Interface (ThermoQuest Finnigan) to decompose CH_4_ into CO_2_. ^13^C/^12^C ratios were determined with a Delta^PLUS^XL mass spectrometer (ThermoQuest Finnigan, Bremen, Germany). High-purity CO_2_ (Messer Griesheim, Frankfurt, Germany) was used as the working monitoring gas. ^13^C/^12^C ratios (δ^13^C values) are expressed in the conventional δ notation in per mil versus VPDB, calculated as:

1 $$\,\delta {}^{13}C_{{VPDB}}=\left(\frac{{\left(\frac{13C}{12C}\right)}_{{Sample}}}{{\left(\frac{13C}{12C}\right)}_{{Standard}}}\right)-1$$

δ^13^C values were corrected using three reference standards of high-purity CH_4_ with δ^13^C values of –54.5 ± 0.2 ‰ (Isometric Instruments, Victoria, Canada), –66.5 ± 0.2 ‰ (Isometric Instruments) and –42.3 ± 0.2 ‰ (in-house), calibrated against International Atomic Energy Agency and NIST reference substances.

### δ^2^H stable isotope measurements (GC-TC-IRMS)

δ^2^H values for CH_4_ were determined using GC-temperature conversion-isotope ratio mass spectrometry (GC-TC-IRMS). The analytical set-up was the same as the one used for δ^13^C stable isotope measurements except that the He flow rate was changed to 0.6 ml min^−1^ and, instead of combustion to CO_2_ and H_2_O, CH_4_ was thermolytically converted (at 1450 °C) to hydrogen and carbon. After IRMS measurements, the obtained δ^2^H values were corrected by using two reference standards of high-purity CH_4_ with δ^2^H values of –149.9‰  ±  0.2‰ (T-iso2, Isometric Instruments) and –190.6‰  ±  0.2‰ (in house). All δ^2^H values are expressed in the conventional δ notation in per mil versus Vienna Standard Mean Ocean Water (VSMOW), calculated as

2 $$\,{{{{{\rm{\delta }}}}}}{}^{2}H_{{VSMOW}}=\left(\frac{{\left(\frac{2H}{1H}\right)}_{{Sample}}}{{\left(\frac{2H}{1H}\right)}_{{Standard}}}\right)-1$$

### Statistics

Unless indicated otherwise, all experiments were performed with *N* = 3 replicates (3 biological replicates). To test for significant differences in CH_4_ formation between two samples, single-factor analysis (two-tailed students *t* test) of variance (ANOVA) was used.

## Supplementary information

Supplementary Information

Peer Review File

## Data Availability

All data are available in the main text or the supplementary information. The data generated in this study have been deposited on the Edmond database^62^, the open repository of the Max Planck Society, under 10.17617/3.6X6JXR.

## References

[1] Badr O, Probert D, O’callaghan PW (1991). Atmospheric methane: Its contribution to global warming. Appl. Energy..

[2] Haqq-Misra JD, Domagal-Goldman SD, Kasting PJ, Kasting JF (2009). A revised, hazy methane greenhouse for the Archean Earth. Astrobiology..

[3] Catling DC, Zahnle KJ (2020). The Archean atmosphere. Sci. Adv..

[4] Feulner G (2012). The faint young Sun problem. Rev. Geophys..

[5] Kasting JF (2014). Atmospheric composition of Hadean–early Archean Earth: The importance of CO. Geol. Soc. Am. Spec. Pap..

[6] Wolfe JM, Fournier GP (2018). Horizontal gene transfer constrains the timing of methanogen evolution. Nat. Ecol. Evol..

[7] Kharecha P, Kasting J, Siefert J (2005). A coupled atmosphere-ecosystem model of the early Archean earth. Geobiology..

[8] Stüeken EE, Buick R (2018). Environmental control on microbial diversification and methane production in the Mesoarchean. Precambrian Res..

[9] Zahnle K, Claire M, Catling AD (2006). The loss of mass-independent fractionation in sulfur due to a Palaeoproterozoic collapse of atmospheric methane. Geobiology..

[10] Zahnle KJ, Gacesa M, Catling DC (2019). Strange messenger: A new history of hydrogen on Earth, as told by Xenon. Geochim. Cosmochim. Acta..

[11] Ernst L (2022). Methane formation driven by reactive oxygen species across all living organisms. Nature..

[12] Bižić M (2020). Aquatic and terrestrial cyanobacteria produce methane. Sci. Adv..

[13] Klintzsch T (2019). Methane production by three widespread marine phytoplankton species: Release rates, precursor compounds, and potential relevance for the environment. Biogeosciences..

[14] Hartmann JF (2020). High spatiotemporal dynamics of methane production and emission in oxic surface water. Environ. Sci. Technol..

[15] Lenhart K (2012). Evidence for methane production by saprotrophic fungi. Nat. Commun..

[16] Keppler F, Hamilton JTG, Braß M, Röckmann T (2006). Methane emissions from terrestrial plants under aerobic conditions. Nature..

[17] Rogers KL, Schulte MD (2012). Organic sulfur metabolisms in hydrothermal environments. Geobiology..

[18] Zeng X, Alain K, Shao Z (2021). Microorganisms from deep-sea hydrothermal vents. Mar. Life sci. Technol..

[19] Duperron S (2019). The bacterial symbionts of closely related hydrothermal vent snails with distinct geochemical habitats show broad similarity in chemoautotrophic gene content. Front. Microbiol..

[20] Zherebker A (2021). Speciation of organosulfur compounds in carbonaceous chondrites. Sci. Rep..

[21] Vogt M, Hopp J, Gail HP, Ott U, Trieloff M (2019). Acquisition of terrestrial neon during accretion—A mixture of solar wind and planetary components. Geochim. Cosmochim. Acta..

[22] Dunbar KL, Scharf DH, Litomska A, Hertweck C (2017). Enzymatic carbon-sulfur bond formation in natural product biosynthesis. Chem. Rev..

[23] Althoff F (2014). Abiotic methanogenesis from organosulphur compounds under ambient conditions. Nat. Commun..

[24] Enami S, Sakamoto Y, Colussi AJ (2014). Fenton chemistry at aqueous interfaces. Proc. Natl. Acad. Sci. USA.

[25] Dodd MS (2022). Abiotic anoxic iron oxidation, formation of Archean banded iron formations, and the oxidation of early Earth. Earth Planet Sci. Lett..

[26] Rush JD, Koppenol WH (1990). Reactions of Fe(II)-ATP and Fe(II)-citrate complexes with t-butyl hydroperoxide and cumyl hydroperoxide. FEBS Lett..

[27] Lueder U, Jørgensen BB, Kappler A, Schmidt C (2020). Photochemistry of iron in aquatic environments. Environ. Sci. Process. Impacts..

[28] Glebov EM (2011). Intermediates in photochemistry of Fe(III) complexes with carboxylic acids in aqueous solutions. Photochem. Photobiol. Sci..

[29] Bruskov VI, Masalimov ZK, Chernikov AV (2002). Heat-induced generation of reactive oxygen species in water. Dokl. Biochem. Biophys..

[30] Chang Y (2020). Water photolysis and its contributions to the hydroxyl dayglow emissions in the atmospheres of Earth and Mars. J. Phys. Chem..

[31] Jin F, Wei M, Liu C, Ma Y (2017). The mechanism for the formation of OH radicals in condensed-phase water under ultraviolet irradiation. Phys. Chem. Chem. Phys..

[32] Azrague K (2005). Hydrogen peroxide evolution during V-UV photolysis of water. Photochem. Photobiol. Sci..

[33] Boyle JW, Ghormley JA, Hochanadel CJ, Riley JF (1969). Production of hydrated electrons by flash photolysis of liquid water with light in the first continuum. J. Phys. Chem..

[34] Agangi A, Hofmann A, Ossa Ossa F, Paprika D, Bekker A (2021). Mesoarchaean acidic volcanic lakes: A critical ecological niche in early land colonisation. Earth Planet Sci Lett..

[35] Timoshnikov VA, Kobzeva TV, Polyakov NE, Kontoghiorghes GJ (2015). Inhibition of Fe^2+^- and Fe^3+^- induced hydroxyl radical production by the iron-chelating drug deferiprone. Free Radic. Biol. Med..

[36] Benckelberg H, Warneck P (1995). Photodecomposition of Iron(III) hydroxo and sulfato complexes in aqueous solution: wavelength dependence of OH and SO_4_-Quantum Yields. J. Phys. Chem..

[37] Krissansen-Totton J, Arney GN, Catling DC (2018). Constraining the climate and ocean pH of the early Earth with a geological carbon cycle model. Proc. Natl. Acad. Sci. U.S.A..

[38] Bernard BB, Brooks JM, Sackett WM (1976). Natural gas seepage in the Gulf of Mexico. Earth Planet. Sci. Lett..

[39] Keller MA, Kampjut D, Harrison SA, Ralser M (2017). Sulfate radicals enable a non-enzymatic Krebs cycle precursor. Nat. Ecol. Evol..

[40] Kitadai N, Kameya M, Fujishima K (2017). Origin of the reductive tricarboxylic acid (rTCA) cycle-type CO_2_ fixation: A Perspective. Life..

[41] Varma SJ, Muchowska KB, Chatelain P, Moran J (2018). Native iron reduces CO_2_ to intermediates and end-products of the acetyl-CoA pathway. Nat. Ecol. Evol..

[42] Chu XY, Xu YY, Tong XY, Wang G, Zhang HY (2022). The legend of ATP: From origin of life to precision medicine. Metabolites..

[43] Kebukawa Y, Asano S, Tani A, Yoda I, Kobayashi K (2022). Gamma-ray-induced amino acid formation in aqueous small bodies in the early solar system. ACS Cent. Sci..

[44] Benzing K, Comba P, Martin B, Pokrandt B (2017). bF. Keppler, Nonheme iron‐oxo‐catalyzed methane formation from methyl thioethers: Scope, mechanism, and relevance for natural systems. Chem. Eur. J..

[45] Bokare AD, Choi W (2014). Review of iron-free Fenton-like systems for activating H_2_O_2_ in advanced oxidation processes. J Hazard. Mater..

[46] Hussain S, Aneggi E, Goi D (2021). Catalytic activity of metals in heterogeneous Fenton-like oxidation of wastewater contaminants: a review. Environ. Chem. Lett..

[47] Foyer CH, Noctor G (2011). Ascorbate and glutathione: The heart of the redox hub. Plant Physiol..

[48] Oikawa S, Kawanishi S (1998). Distinct mechanisms of site-specific DNA damage induced by endogenous reductants in the presence of iron(III) and copper(II). Biochim. Biophys. Acta Gene Regul. Mech..

[49] Lueder U, Jorgensen B, Maisch M, Schmidt C, Kappler A (2022). Influence of Fe(III) source, light quality, photon flux and presence of oxygen on photoreduction of Fe(III)-organic complexes – Implications for light-influenced coastal freshwater and marine sediments. Sci. Total Environ..

[50] Russell MJ, Nitschke W (2017). Methane: Fuel or exhaust at the emergence of life?. Astrobiology..

[51] Nitschke W, Russell MJ (2013). Beating the acetyl coenzyme A-pathway to the origin of life. Philos. Trans. R. Soc. Lond. B, Biol. Sci..

[52] Ettwig KF (2010). Nitrite-driven anaerobic methane oxidation by oxygenic bacteria. Nature..

[53] Adam PS, Kolyfetis GE, Bornemann TLV, Vorgias CE, Probst AJ (2022). Genomic remnants of ancestral methanogenesis and hydrogenotrophy in Archaea drive anaerobic carbon cycling. Sci. Adv..

[54] Ordóñez C (2023). Evaluation of the methane paradox in four adjacent pre-alpine lakes across a trophic gradient. Nat. Commun..

[55] Canfield DE (1998). A new model for Proterozoic ocean chemistry. Nature..

[56] Canfield DE (2008). Ferruginous conditions dominated later neoproterozoic deep-water chemistry. Science..

[57] Lassen C, Ploug H, Jørgensen BB (1992). A fibre-optic scalar irradiance microsensor: application for spectral light measurements in sediments. FEMS Microbiol. Lett..

[58] Klatt JM (2015). Anoxygenic photosynthesis controls oxygenic photosynthesis in a cyanobacterium from a sulfidic spring. Appl. Environ. Microbiol..

[59] S. S. Nielsen, C. E. Carpenter, R. E. Ward Eds., Iron Determination by Ferrozine Method. *Food analysis laboratory manual*. 157–159 (Springer, 2017).

[60] Vitt S (2014). The F420-reducing [NiFe]-Hydrogenase complex from *Methanothermobacter marburgensis*, the first X-ray structure of a group 3 family member. J. Mol. Biol..

[61] Ousley S, de Beer D, Bejarano S, Chennu A (2022). High-resolution dynamics of hydrogen peroxide on the surface of scleractinian corals in relation to photosynthesis and feeding. Front Mar. Sci..

[62] Rebelein, J. G. Methane formation driven by light and heat prior to the origin of life and beyond. *Edmond*, 10.17617/3.6X6JXR. (2023). 10.1038/s41467-023-39917-0 PMC10394037 37528079

