# Supplementary material for: Methane formation driven by light and heat prior to the origin of life and beyond
Source: Nat Commun. 2023 Aug 1;14:4364. doi: 10.1038/s41467-023-39917-0 (PMC10394037; doi:10.1038/s41467-023-39917-0)
Supplement: Supplementary file 1 — Supplementary Information [file 41467_2023_39917_MOESM1_ESM.pdf]

# Supplementary Information

## Methane formation driven by light and heat prior to the origin of life and beyond

Leonard Ernst[1,2] *, Uladzimir Barayeu[3,4], Jonas Hädeler[5], Tobias P. Dick[3,4], Judith M. Klatt[2,6,7], Frank Keppler[5,8] & Johannes G. Rebelein[1,2] *

[1] Max-Planck-Institute for Terrestrial Microbiology; 35043 Marburg, Germany.
[2] Center for Synthetic Microbiology (SYNMIKRO); 35032 Marburg, Germany.
[3] Division of Redox Regulation, German Cancer Research Center (DKFZ), DKFZ-ZMBH Alliance; 69120 Heidelberg, Germany.
[4] Faculty of Biosciences, Heidelberg University; 69120 Heidelberg, Germany.
[5] Institute of Earth Sciences, Heidelberg University; 69120 Heidelberg, Germany.
[6] Microcosm Earth Center, Max Planck Institute for Terrestrial Microbiology & Philipps University Marburg; 35032 Marburg, Germany.
[7] Biogeochemistry Group, Department for Chemistry, Philipps University Marburg; 35032 Marburg, Germany.
[8] Heidelberg Center for the Environment HCE, Heidelberg University; 69120 Heidelberg, Germany.

* *To whom correspondence should be addressed:* leonard.ernst@mpi-marburg.mpg.de (L.E.); johannes.rebelein@mpi-marburg.mpg.de (J.G.R.)

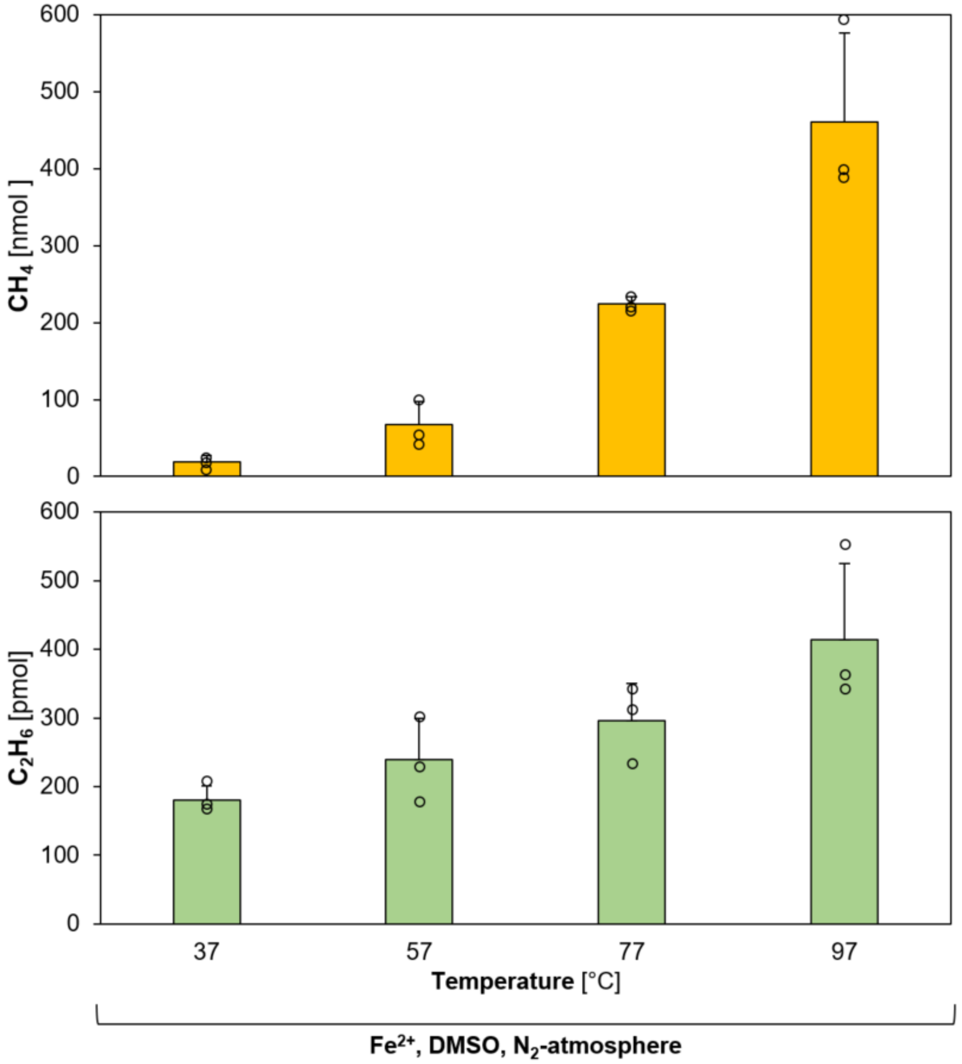


**Supplementary Fig. 1. Heat-driven $CH_4$ and $C_2H_6$ formation from DMSO.** Formed $CH_4$ amounts increase from ~20 nmol (37 °C) to ~460 nmol (97 °C). Formed $C_2H_6$ amounts increase from ~190 pmol (37 °C) to ~415 pmol (97 °C), thereby resulting in $CH_4$:$C_2H_6$ ratios from ~110 (37 °C) to ~1100 (97 °C). In a total volume of 4 mL, samples containing a 20 mM potassium phosphate buffer (pH 7), 1 M DMSO, 10 mM $FeSO_4$ were incubated at different temperatures in sealed 20 mL glass vials under $N_2$ for 4 days. The bars are the mean + standard deviation of triplicates, shown as circles.

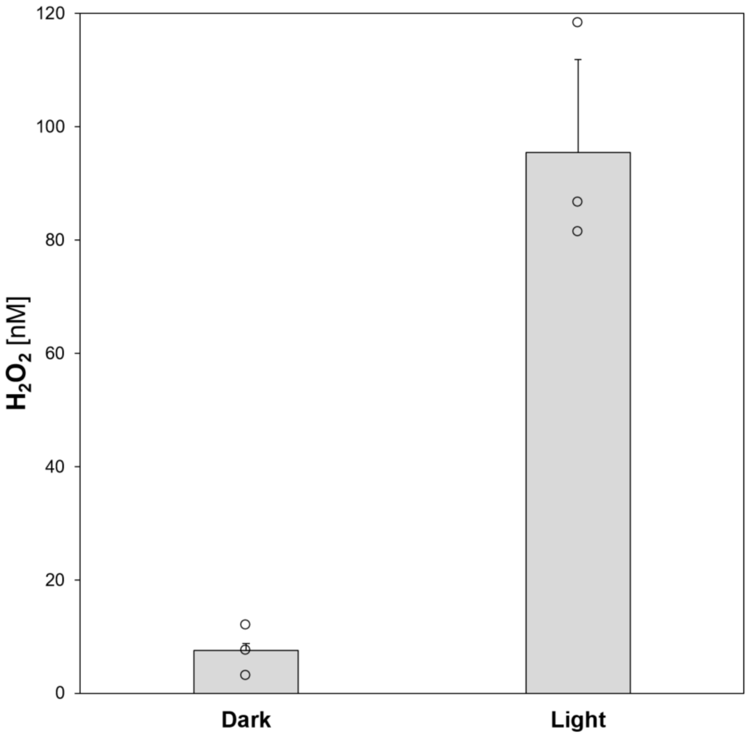


**Supplementary Fig. 2. Light-driven $H_2O_2$ formation in pure buffer.** Formed $H_2O_2$ concentrations increase from ~7 nM in the dark to ~95 nM under light. In a total volume of 4 mL, samples contained 20 mM potassium phosphate buffer (pH 7) that was bubbled with $N_2$ for 1 h and kept in an anoxic tent for 5 days. Samples were incubated in the dark or under illumination (Supplementary Fig. 3) for 20 h and analysed via fluorescence-based $H_2O_2$ endpoint measurements (see Methods). The bars are the mean + standard deviation of triplicates, shown as circles.

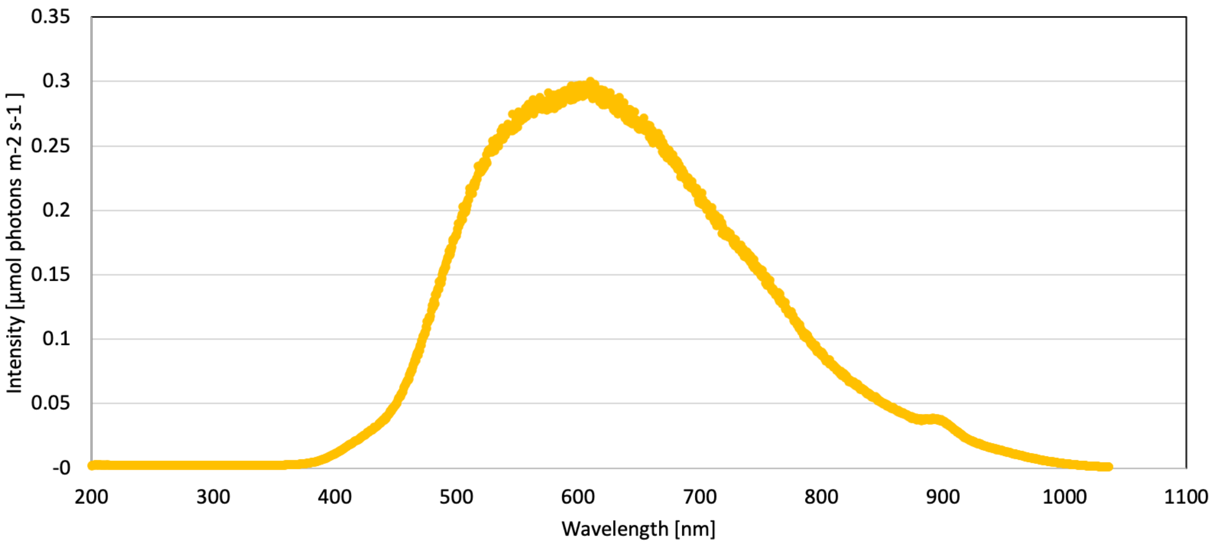


**Supplementary Fig. 3. Light spectrum of the used light bulbs.** For broad-spectrum sample illumination, Osram (Superlux, Super E SIL 60) light bulbs were used, with an intensity of 82 ± 4 µmol photons $m^{-2}$ $s^{-1}$, and an energy flux of 52 ± 2 kJ $m^{-2}$ $h^{-1}$. The light spectrum was determined with a spectrometer (see Methods).

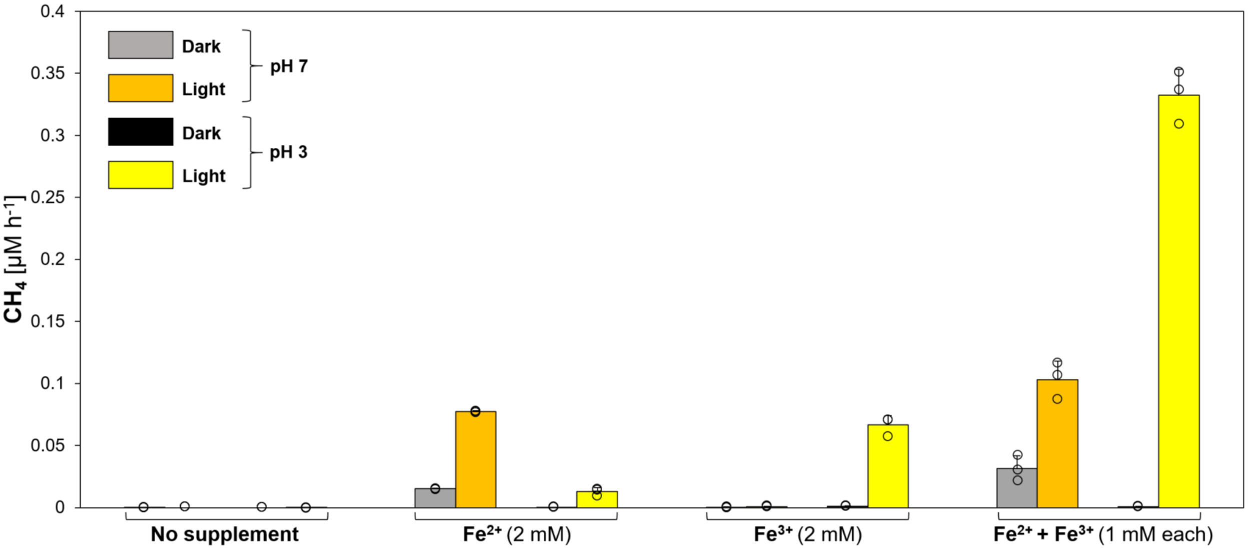


**Supplementary Fig. 4. Light-driven $CH_4$ formation is enhanced upon $[Fe(H_2O)_6]^{3+}$ photolysis under acidic conditions.** Only trace $CH_4$ levels (<0.0002 µM $h^{-1}$) were measured without iron addition. Upon $Fe^{2+}$-supplementation, $CH_4$ formation rates increased at pH 7 from ~0.015 µM $h^{-1}$ in the dark to ~0.077 µM $h^{-1}$ under light and at pH 3 from <0.0002 µM $h^{-1}$ in the dark to ~0.013 µM $h^{-1}$ under light, suggesting a light-driven ·OH formation from $OH^-$. Conversely, ~0.67 µM $CH_4$ $h^{-1}$ are formed upon $Fe^{3+}$-supplementation in the light under acidic conditions, while only trace amounts of $CH_4$ were detected under pH-neutral conditions or in the dark. At pH 7, a stoichiometric 1:1 ratio of $Fe^{3+}$ and $Fe^{2+}$ increased $CH_4$ formation rates by ~1.3-fold to ~0.1 µM $h^{-1}$ in comparison to $Fe^{2+}$-supplemented samples, while the corresponding $CH_4$ formation rates at pH 3 increased ~25-fold to ~0.316 µM $h^{-1}$. Samples consisting of buffered solutions (pH 3 or 7) were incubated in the dark or under broad spectrum light (Supplementary Fig. 3) for 24 h under $N_2$. The bars are the mean + standard deviation of triplicates, shown as circles.

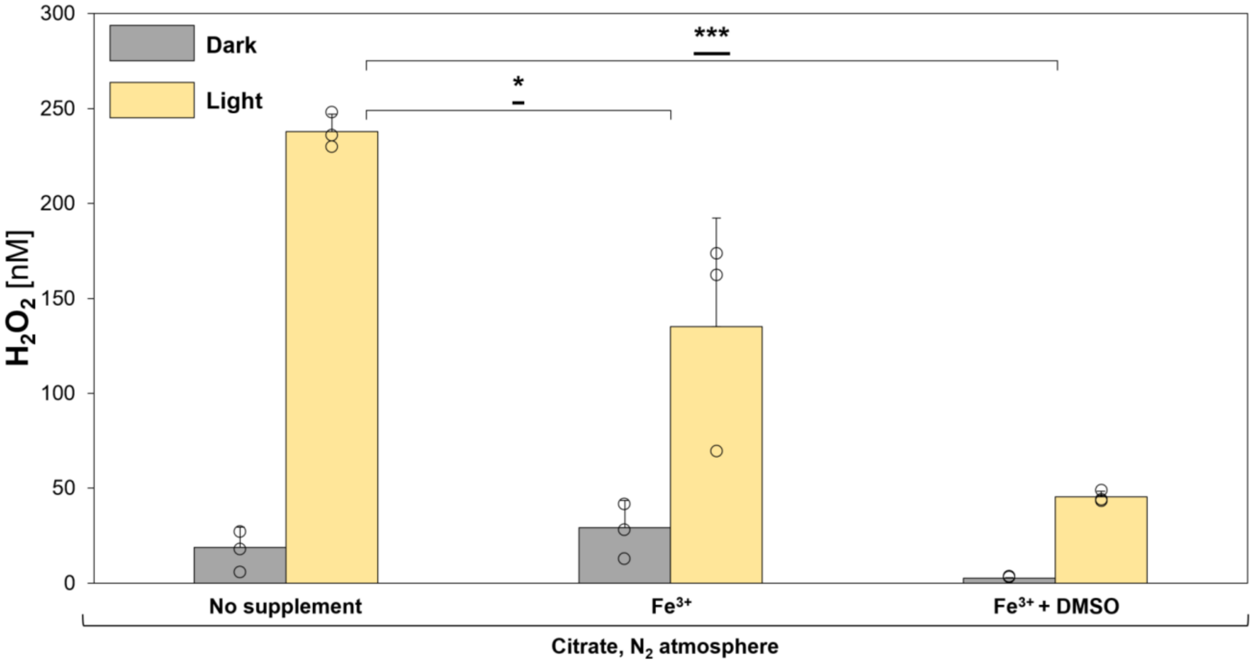


**Supplementary Fig. 5. Iron and DMSO reduce $H_2O_2$ concentrations generated by light.** Under light, $H_2O_2$ concentrations decrease from ~238 nM (buffer + citrate) to ~135 nM in the presence of iron (buffer + citrate + $Fe^{3+}$). This decrease in $H_2O_2$ concentration indicates the reaction of $H_2O_2$ with $Fe^{2+}$ formed by LMCT. Upon addition of DMSO, $H_2O_2$ levels further decrease to ~45 nM under light, underlining the ROS-scavenging effect of DMSO. In a total volume of 4 mL, samples contained 20 mM potassium phosphate buffer (pH 7), 10 mM citrate and, optionally, 2 mM $FeCl_3$ and 500 mM DMSO that were previously degassed with $N_2$ and kept in an anoxic tent overnight. Samples were incubated in the dark or under illumination (Supplementary Fig. 3) for 3 h and directly analysed via fluorescence-based $H_2O_2$ endpoint measurements (see Methods). Statistical analysis was performed using paired two-tailed *t*-tests, *: $p \leq 0.05$, ***: $p \leq 0.001$. The bars are the mean + standard deviation of triplicates, shown as circles.

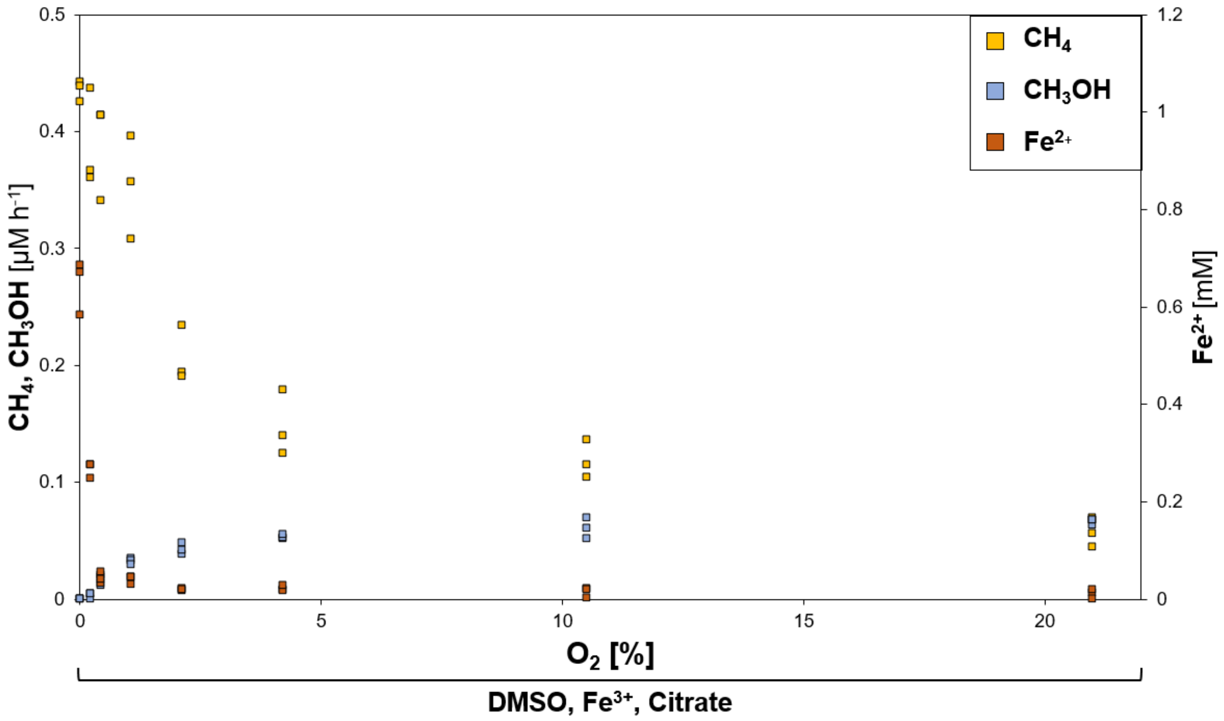


**Supplementary Fig. 6. Light-driven formation of $CH_4$, $CH_3OH$ and $Fe^{2+}$ from DMSO and $Fe^{3+}$ under varying $O_2$ concentrations.** In a total volume of 4 mL, samples containing 20 mM potassium phosphate buffer (pH 7), 500 mM DMSO, 2 mM $FeCl_3$ and 10 mM citrate were incubated for one day in sealed 20 mL glass vials under constant broad-spectrum illumination (Supplementary Fig. 3). Mixtures of air and $N_2$ were generated and adjusted to 1 bar, ranging from ~21 % $O_2$ to pure $N_2$ (0 % $O_2$). From 0 % $O_2$ to ~1 % $O_2$, $CH_4$ rates decreased from ~0.44 µM $h^{-1}$ to ~0.35 µM $h^{-1}$, while $Fe^{2+}$ levels decreased from ~1 mM to ~0.07 mM. From ~1 % $O_2$ to 21 % $O_2$, $CH_4$ rates further decreased by an additional ~0.2 µM $h^{-1}$, while $Fe^{2+}$ levels further decreased from ~0.07 mM to ~0.04 mM, indicating an immediate $Fe^{2+}$ oxidation by $O_2$ or the Fenton reaction at high $O_2$ concentrations and a formation of excess $Fe^{2+}$ under suboxic and anoxic conditions. Apart from the anoxic sample (0 % $O_2$), formation of $CH_3OH$ was detected in the presence of $O_2$, ranging from ~0.003 µM $h^{-1}$ (~0.2 % $O_2$) to ~0.07 µM $h^{-1}$ (21 % $O_2$). Experiments were conducted in triplicates; individual values are shown.

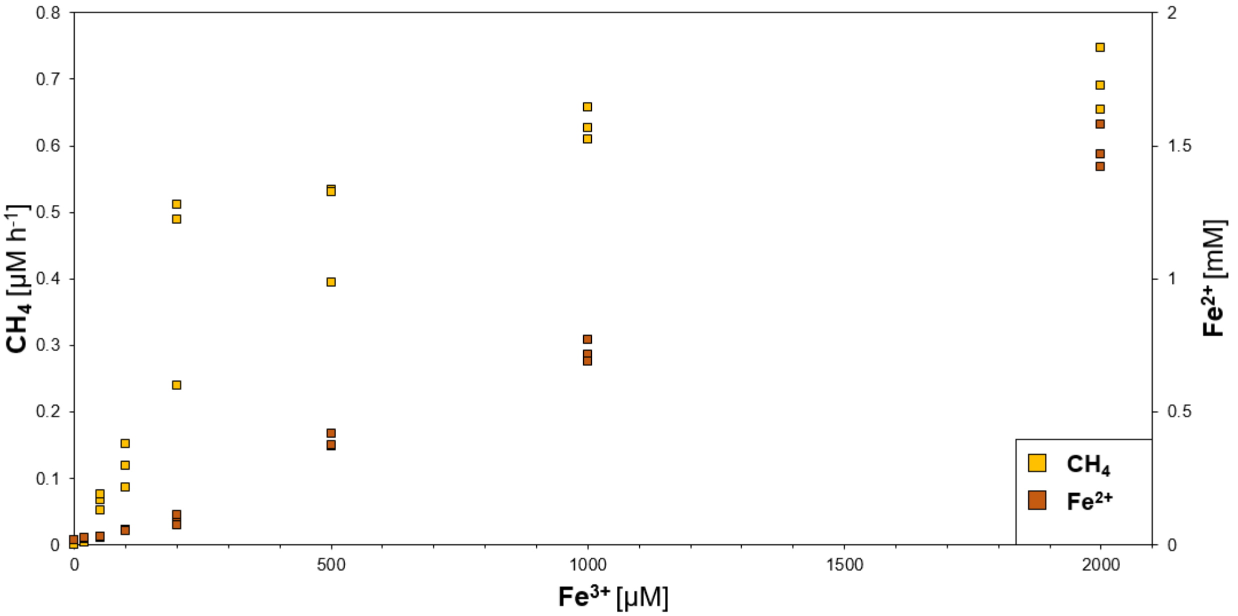


**Supplementary Fig. 7. Light-driven $CH_4$ formation from DMSO correlates with the iron concentration.** In a total volume of 4 mL, samples containing 20 mM potassium phosphate buffer (pH 7), 500 mM DMSO and 10 mM citrate were incubated for one day under $N_2$ in sealed 20 mL glass vials under constant broad-spectrum illumination (Supplementary Fig. 3). Different amounts of $Fe^{3+}$ (as $FeCl_3$) were added, ranging from 0 µM to 2000 µM. Only trace amounts of $CH_4$ (<0.01 µM $h^{-1}$) were formed upon supplementation with 0 or 20 µM $Fe^{3+}$. Methane amounts then increased from ~0.06 µM $h^{-1}$ $CH_4$ (50 µM $Fe^{3+}$) to ~0.4 µM $h^{-1}$ (200 µM $Fe^{3+}$), with $Fe^{2+}$ levels also increasing from ~0.03 mM to ~0.11 mM. From 200 µM $Fe^{3+}$ to 2000 µM $Fe^{3+}$ supplementation, $CH_4$ levels increased by ~0.3 µM $h^{-1}$. In the same range, $Fe^{2+}$ levels increased from ~0.11 mM to ~1.5 mM, indicating that LMCT-driven $Fe^{2+}$ formation occurred at higher rates as its consumption to $Fe^{3+}$ via the photochemical Fenton reaction. Experiments were conducted in triplicates; individual values are shown.

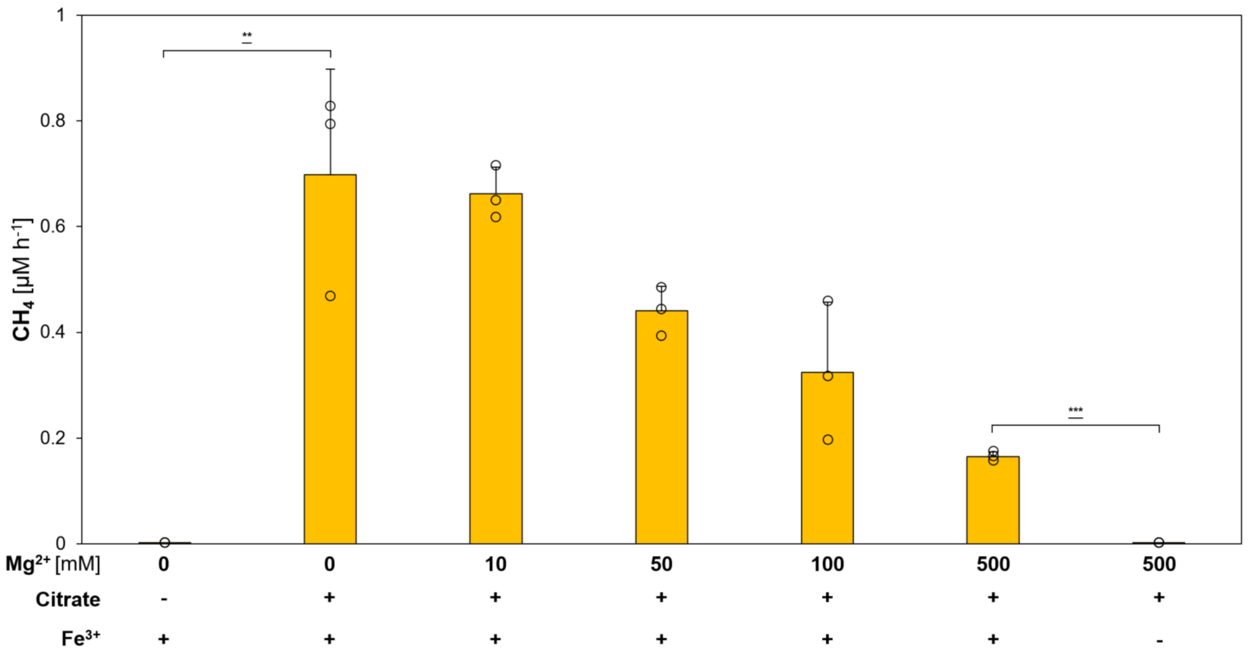


**Supplementary Fig. 8. Magnesium decreases light-driven $CH_4$ formation from DMSO.** In a total volume of 4 mL, samples containing 20 mM potassium phosphate buffer (pH 7), 500 mM DMSO and, optionally, 2 mM $Fe^{3+}$ (as $FeCl_3$) and 10 mM citrate were incubated for one day under $N_2$ in sealed 20 mL glass vials under constant broad-spectrum illumination (Supplementary Fig. 3). Different amounts of $Mg^{2+}$ (as $MgCl_2$) were added, ranging from 0 mM to 500 mM. Only trace amounts of $CH_4$ (<0.01 µM $h^{-1}$) were formed in the presence of $Mg^{2+}$ and citrate Upon supplementation with $Fe^{3+}$ without addition of citrate trace amounts of $CH_4$ (<0.01 µM $h^{-1}$) were detected. Upon citrate and $Fe^{3+}$ supplementation, $CH_4$ rates increased to ~0.7 µM $h^{-1}$, while increasing $Mg^{2+}$ amounts decreased $CH_4$ rates until ~0.16 µM $h^{-1}$ at 500 mM $Mg^{2+}$. Experiments were conducted in triplicates; individual values are shown as cirlces.

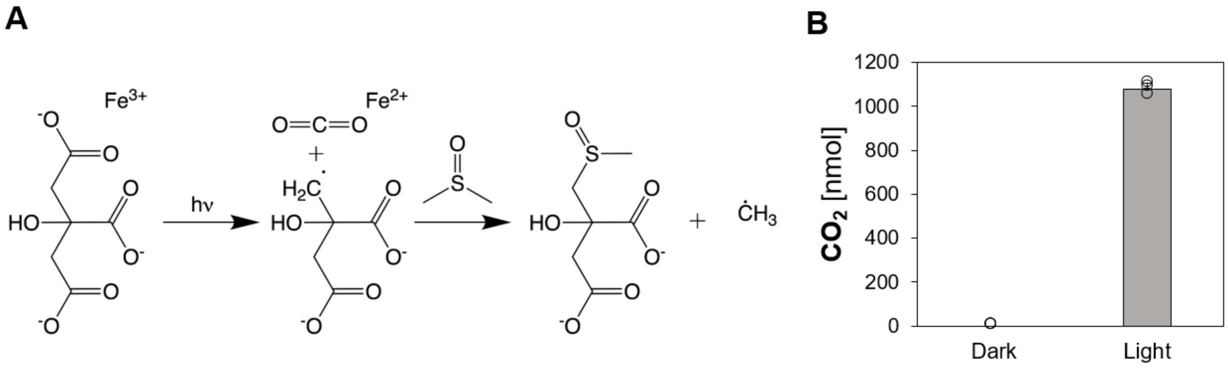


**Supplementary Fig. 9. Decomposition of the LMCT-induced citrate radical drives ·$CH_3$ formation from DMSO. (A)** Illumination of a $Fe^{3+}$-citrate complex results in $Fe^{2+}$ and a citrate radical, disassembling into $CO_2$ and a carbon-centered radical that further reacts with DMSO by cleaving off a ·$CH_3$. **(B)** While no $CO_2$ is formed in the dark, ~1080 nmol $CO_2$ is detected from illuminated samples containing 2 mM $FeCl_3$, 10 mM citrate, 500 mM DMSO in 20 mM potassium phosphate buffer (pH 7) under $N_2$, after 24 h. The bars are the mean + standard deviation of three independent measurements, shown as circles.

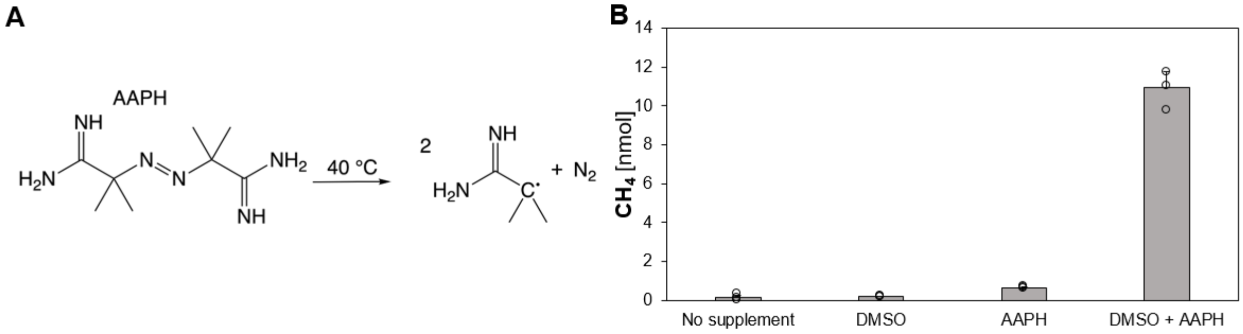


**Supplementary Fig. 10. Organic radicals drive $CH_4$ formation from DMSO. (A)** At 40 °C, the compound 2,2′-azobis(2-methylpropionamidine) dihydrochloride (AAPH) decomposes into 2 carbon-centered organic radicals and $N_2$. **(B)** $CH_4$ is formed upon the interaction between DMSO and AAPH. While only ~0.2 nmol or ~0.6 nmol $CH_4$ are formed from DMSO or AAPH alone, respectively, ~11 nmol $CH_4$ are formed upon combining both substances, indicating $CH_4$ formation driven by carbon-centered radicals. 500 mM DMSO was mixed with 20 mM AAPH and incubated for 3 h in $N_2$-saturated DPBS buffer at 40°C under $N_2$. The bars are the mean + standard deviation of triplicates, shown as circles.

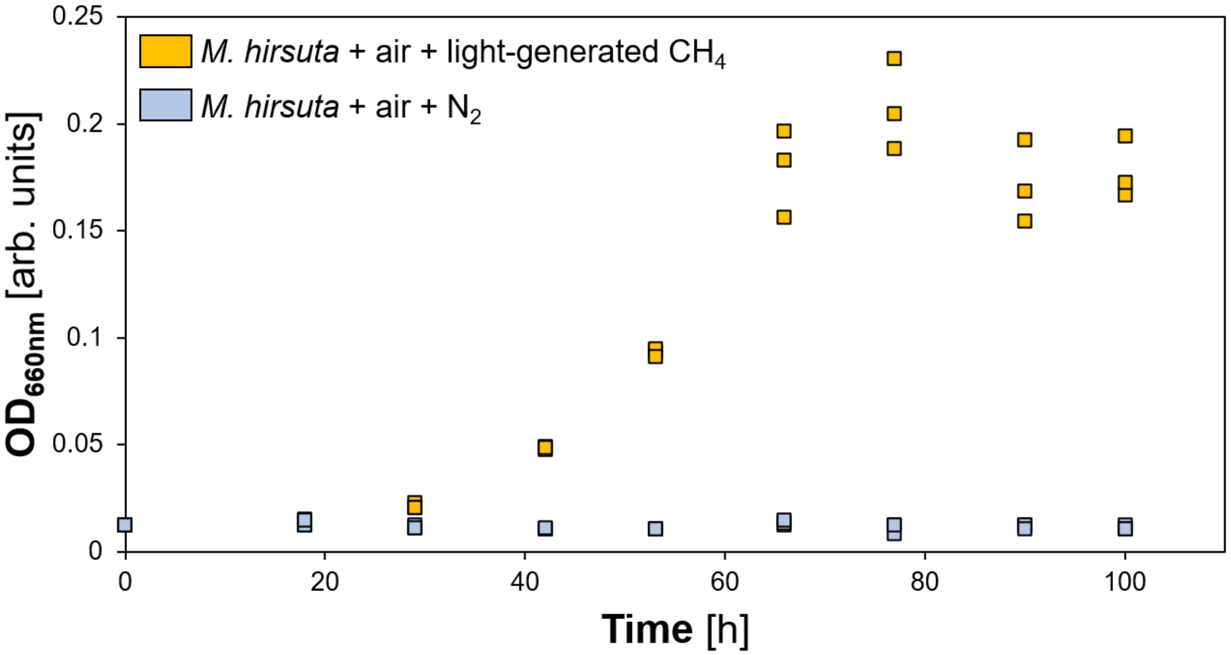


**Supplementary Fig. 11. Light-driven $CH_4$ formation sustains methanotrophic growth of *Methylocystis hirsuta*.** Methanotrophic growth of *M. hirsuta* is sustained by light-generated $CH_4$. While $CH_4$-supplemented samples (yellow squares) grew, $N_2$-supplemented samples (blue squares) did not exhibit bacterial growth (see Methods). Experiments were conducted in triplicates; individual values are shown.
